# Identification of histone acetyltransferase genes responsible for cannabinoid synthesis in hemp

**DOI:** 10.1186/s13020-023-00720-0

**Published:** 2023-02-13

**Authors:** Yufei Cheng, Kang Ning, Yongzhong Chen, Cong Hou, Haibin Yu, Huatao Yu, Shilin Chen, Xiaotong Guo, Linlin Dong

**Affiliations:** 1 Key Laboratory of Beijing for Identification and Safety Evaluation of Chinese Medicine, Institute of Chinese Materia Medica, China Academy of Chinese Medical Sciences, Beijing, 100700 China; 2 College of Agronomy, Ludong University, Yantai, 264000 China; 3 Yunnan Hemp Industrial Investment CO.LTD, Kunming, 650217 China

**Keywords:** Histone acetyltransferase, Hemp, Acetylation, Cannabinoid, Abiotic stress

## Abstract

**Background:**

Histone acetyltransferases (HATs) play an important role in plant growth and development, stress response, and regulation of secondary metabolite biosynthesis. Hemp (*Cannabis sativa* L.) is famous for its high industrial, nutritional, and medicinal value. It contains non-psychoactive cannabinoid cannabidiol (CBD) and cannabinol (CBG), which play important roles as anti-inflammatory and anti-anxiety. At present, the involvement of HATs in the regulation of cannabinoid CBD and CBG synthesis has not been clarified.

**Methods:**

The members of *HAT* genes family in hemp were systematically analyzed by bioinformatics analysis. In addition, the expression level of HATs and the level of histone acetylation modification were analyzed based on transcriptome data and protein modification data. Real-time quantitative PCR was used to verify the changes in gene expression levels after inhibitor treatment. The changes of CBD and CBG contents after inhibitor treatment were verified by HPLC-MS analysis.

**Results:**

Here, 11 *HAT* genes were identified in the hemp genome. Phylogenetic analysis showed that hemp *HAT* family genes can be divided into six groups. Cannabinoid synthesis genes exhibited spatiotemporal specificity, and histones were acetylated in different inflorescence developmental stages. The expression of cannabinoid synthesis genes was inhibited and the content of CBD and CBG declined by 10% to 55% in the samples treated by HAT inhibitor (PU139). Results indicated that *CsHAT* genes may regulate cannabinoid synthesis through altering histone acetylation.

**Conclusions:**

Our study provides genetic information of HATs responsible for cannabinoid synthesis, and offers a new approach for increasing the content of cannabinoid in hemp.

**Supplementary Information:**

The online version contains supplementary material available at 10.1186/s13020-023-00720-0.

## Introduction

Post-translational modifications (PTMs) are commonly found in plants and play important roles in many cellular processes, including protein degradation, signalling processes, regulation of gene expression, and protein-protein interactions [1]. For example, ubiquitination plays an important role in the protein degradation, whereas phosphorylation is involved in signal transduction during plant immunity [2, 3]. Protein acetylation is a reversible post-translational modification, which is regulated by histone acetyltransferases (HATs) and deacetylases (HDACs). Histone acetylation/deacetylation is essential for the epigenetic regulation of diverse biological processes, including environmental stress responses in plants. A recent study has shown that histone lysine acetylation and acylation are regulated by environmental and metabolic cues in rice [4]. The first HAT was reported in 1995 and has been since identified in plants, animals, and fungi [5]. Based on their structural characteristics, plant HATs can be divided into four families: HAC, HAF, HAG, and HAM. The HAG family containing HAG1, HAG2 and HAG3 were found in *Arabidopsis* [6]*.* HAT is closely related to plant growth, development, and stress responses [7]. *Arabidopsis* HAT, *AtGCN5*, is necessary for light regulation, and it is also an important factor in maintaining root stem cells [8, 9]. In addition, *AtMYST,* another HAT, regulates the formation and development of male and female gametophytes in *Arabidopsis*. [10] Some studies suggest that the rice HAT, *GW6a*, and ubiquitination receptors can jointly regulate the grain size of rice seeds [11]. GNAT-MYST family HAT genes (*HvMYST*, *HvELP3,* and *HvGCN5*) of barley are expressed at various stages of seed development [12]. Some studies showed that HAT is highly involved in stress responses, such as drought, plant hormones, salt stress, and low temperature. The expressions of *OsHAC703*, *OsHAG703,* and *OsHAM701* are significantly induced by drought stress and ABA [13]. *ZmHATB* and *ZmGCN5* increase after NaCl treatment, accompanied by an upregulation in the global acetylation levels of histones, H3K9, and H4K5 [14]. *AtGCN5* may be involved in the low-temperature stress response modulated by CBF1 [15]. Moreover, HAT member is involved in biotic stress. For example, *CfGCN5* has a regulatory effect on the tea-oil tree, anthracnose [16]. In addition, HAT plays a key role in secondary metabolite biosynthesis. For instance, HATs are critical factors related to production of secondary metabolites in *Aspergillus nige* [17]. Anti-cancer effect of *ganoderma* triterpenes might be related to histone acetylation by regulating GCN5 [18]. HATs are required for the expression of genes involved in phenylpropanoid/benzenoid volatile organic compound biosynthesis and emission [19]. HATs are widely reported because of their remarkable effects.

Hemp (*Cannabis sativa* L.), which originated in Central Asia, is an annual herb belonging to the *Cannabaceae* family [20]. To date, more than 100 cannabinoids have been reported, including tetrahydrocannabinol (THC), cannabidiol (CBD), and cannabigerol (CBG) [21]. Hemp can be classified into two types according the content of THC: drug type (THC > 0.3%) and non-drug type (THC < 0.3%) [22]. Δ9-THC is a psychoactive ingredient. It has addictive properties (sensory perception, motivational effects, analgesic effects, and sleep during abstinence/mood disorders) and other pharmacological effects (cardiovascular response, hormone release, reproductive function, immune regulation, and motor function) [23]. CBD, one of many naturally occurring compounds in cannabis, is a non-psychoactive compound that has become increasingly popular for the treatment of vairous medical ailments. It plays a key role in treating anxiety, insomnia, and epilepsy. [24–26] In addition, CBD can reduce psychotic symptoms and improve quality of life in patients with Parkinson’s disease. [27] CBD, a PPAR-γ receptor agonist, had been speculated to potentially limit the onset of late-onset pulmonary fibrosis in COVID19-recovered patients. [28] CBG is also a non-psychoactive compound produced during the non-enzymatic decarboxylation of cannabinolic acid, which is a key compound in the process of biosynthesis of phytocannabinoid. CBG exhibits a wide range of effects, inter alia, anticancer, antibacterial, and neuromodulatory effects [29–31]. At present, HATs play a key role in the synthesis of phenylpropanoid, *ganoderma* triterpenoid, indole alkaloids, polyketides, and other secondary metabolites [17–19]. The biosynthetic pathway of cannabinoid has been elucidated [32], but the involvement of HATs in the regulation of cannabinoid synthesis has not been clarified. We have hypothesized that HATs could be involved in the regulation of cannabinoid synthesis in hemp. Thus, the identification and functional analysis of HATs provide significant information for regulating cannabinoid biosynthesis in hemp.

Herein, we built a multi-omics atlas of hemp and found that protein acetylation occurs widely across cannabinoid biosynthesis enzymes during hemp inflorescence development (unpublished). In this study, we identified and characterized the members of the HAT gene family in hemp and comprehensively analyzed their phylogenetic relationships, structures, chromosomal locations, and expression patterns. We further confirmed that the expression of *CsHAT* genes, cannabinoid synthesis genes, and cannabinoid content changed after treatment with HAT inhibitor (PU139) to confirm the *CsHAT* genes regulating the cannabinoid biosynthesis. These studies are helpful in the analysis of genetic mechanisms underlying candidate *HAT* genes responsible for cannabinoid biosynthesis and the breeding of hemp varieties with high CBD and low THC. Our results offer useful information on abiotic stresses affecting the biosynthesis of secondary metabolites in hemp.

## Materials and methods

### Identification of the CsHAT family

The Hemp genome database, CS10 (https://www.ncbi.nlm.nih.gov/genome/11681?genome_assembly_id=897706), was used to identify the *CsHAT* family. BLASTP searches (*e*-value = 1*e*-5) were used to identify putative *CsHATs*. The Pfam (http://pfam.xfam.org/) and SMART (http://smart.embl-heidelberg.de/) databases were used to confirm the conserved domains of the putative *CsHATs*. ExPASy (http://web.expasy.org/protparam/) and CELLO (http://cello.life.nctu.edu.tw/) were used to predict physicochemical properties and subcellular localization of the hemp HAT gene family.

### Phylogenetic analysis of the CsHAT family

The HATs of *Arabidopsis* and rice were downloaded [33]. The HAT protein sequences of hemp, *Arabidopsis,* and rice were in the FASTA format. These sequences were aligned with ClustalW, and a phylogenetic tree was constructed according to the neighbor-joining method with 1000 bootstrap replicates in MEGA 7.0.

### Motif analysis and Cis-acting element prediction

The Multiple Em for Motif Elicitation (MEME) online tool (http://alternate.meme-suite.org/tools/meme) was used to analyze protein motifs, with a maximum selection of 20 motifs. Upstream sequences (2000 bp) of all *CsHATs* were obtained from the genomic data and analyzed using the PlantCARE online tool (http://bioinformatics.psb.ugent.be/webtools/plantcare/html/). The identified *cis*-acting elements were classified by their functions and visualized using TBtools software.

### Chromosomal location and synteny

Based on the genomic data (CS10), the chromosome location of each *CsHAT* was identified and visualized using the MapChart software. Synteny analysis was performed for *CsHATs* using TBtools software based on the genome information files of *Arabidopsis*, grape, and maize with the following parameters: CPU for BlastP: 6, e-value: 1e-10, and Num of BlastHits: 5.

### Plant materials and inhibitor treatments

A cultivated hemp variety (Yunma 8) was grown in an artificial climate chamber with 14 h light/10 h dark at 20 ± 2 °C until the appearance of an inflorescence (approximately four months). The inhibitor was a freshly prepared working solution of 5 μM PU139 in DMSO. Sample processing was performed as described by Aquea et al. [34], with minor modifications. Briefly, inhibitors were sprayed on the inflorescence and collected at 0, 3, 24, and 72 h after treatment. All collected samples were frozen in liquid nitrogen immediately after removal from the plants and stored at − 80 °C for further experiments.

### RNA extraction and real-time quantitative PCR analysis

The inflorescences were ground into powder in liquid nitrogen. RNA was extracted using the Quick RNA Isolation Kit (Waryoung, China), following the manufacturer’s instructions, and all RNA samples were analyzed by agarose gel electrophoresis. cDNA was synthesized using the FastQuant RT Kit (Tiangen, China). qRT-PCR analysis was performed using a SLAN-96P real-time PCR system (SLAN, China), whereby each gene was analyzed in three biological replicates and three technical replicates using StarLighter SYBR Green qPCR Mix (Forever Star, Beijing). *CsEF1α* was used as an internal reference [35]. The reaction was run using the following program: 95 °C for 3 min, followed by 40 cycles of 95 °C for 10 s and 60 °C for 20 s. Fold changes were calculated using the 2^−ΔΔCt^ method [36]. The primer sequences used in this study are listed in Additional file 1: Table S1.

### Measurement of CBD and CBG contents

Frozen inflorescences were ground into a powder in liquid nitrogen; then, 0.05 g was accurately weighed, and 5 mL 90% methanol was added. The samples were mixed by vortexing. The resultant mixture was incubated overnight at 4 °C, extracted by ultrasonication for 30 min, and centrifuged at 12000 rpm for 10 min. The supernatant was collected and used to assay CBD and CBG contents on Agilent 1290 liquid chromatograph and Agilent 6410 triple quadrupole mass spectrometer. A C18 column (3.0 mm × 100 mm, 1.8 μm) was used for chromatographic separation at 30 °C. Mobile phases A (0.1% formic acid in water) and B (0.1% formic acid in acetonitrile) were used for separation under the following gradient elution program: 0–14 min, 70%–80% B; 14–17 min, 80–100% B; 17–20 min, 100% B. The flow rate was 0.25 mL/min, and 1 μL of the sample was injected. The experiment was repeated thrice. The acquisition parameters for each compound are listed in Additional file 2: Table S2.

## Results

### Identification and characterization of HAT genes in hemp

**Fig. 1:**
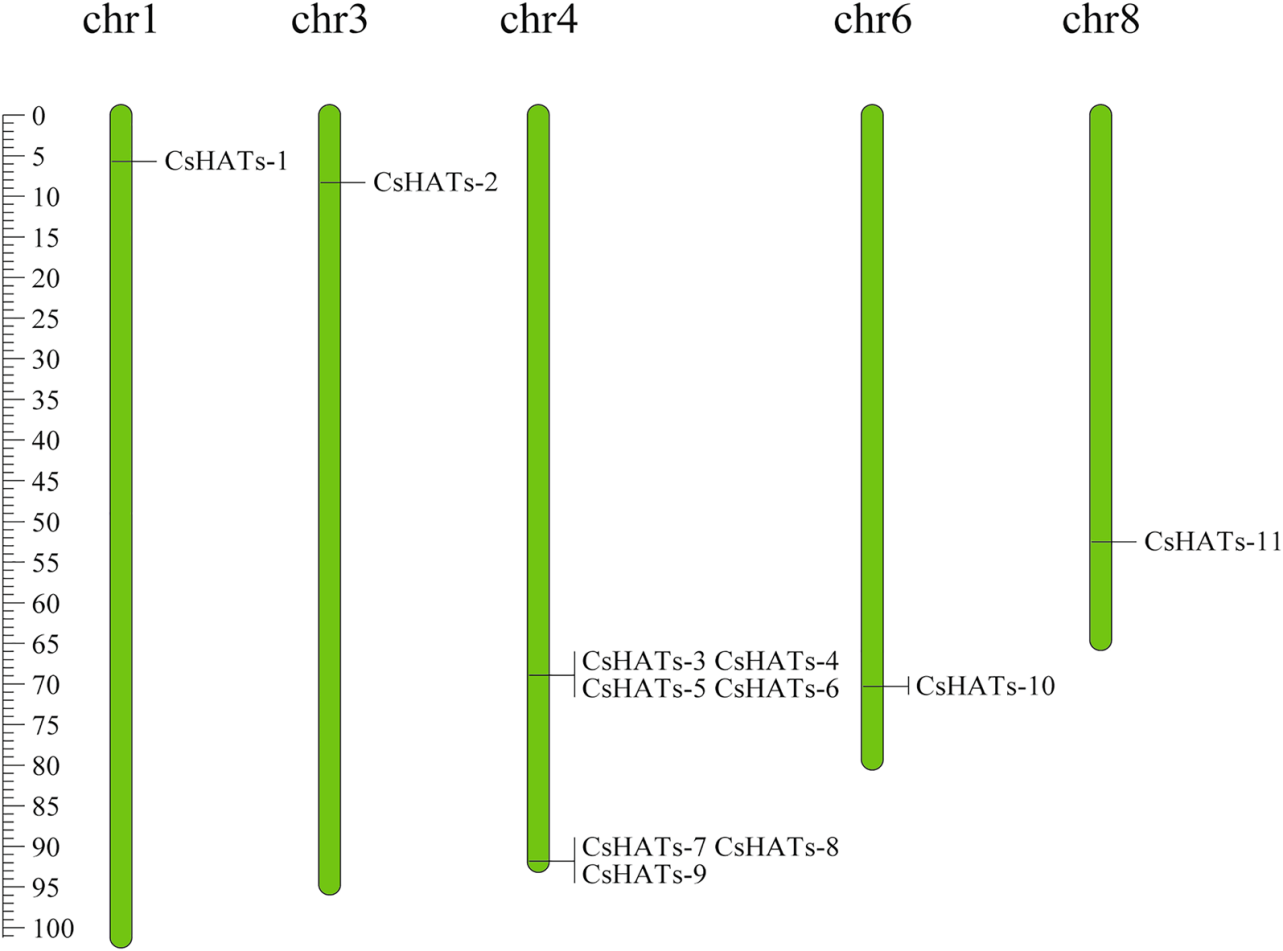
Chromosomal location of 11 histone acetyltransferase genes (*HATs*) in hemp

On the basis of the BLASTP, Pfam, and SMART results, 11 acetyltransferase genes were identified in the hemp genome (Table 1). We named the family members, *CsHATs-1*–*CsHATs-11*, according to their chromosomal location. Eleven *CsHAT* genes were unevenly distributed on chr1, chr3, chr4, chr6, and chr8 (Fig. 1). Seven *CsHAT* genes (*CsHATs-3*, *CsHATs-4*, *CsHATs-5*, *CsHATs-6*, *CsHATs-7*, *CsHATs-8*, and *CsHATs-9*) were located on chr4, harboring the largest number of genes. *CsHATs-1*, *CsHATs-2*, *CsHATs-10*, and *CsHATs-11* were located on chr1, chr3, chr6, and chr8, respectively.

**Table 1:** Detailed information of 11 predicted HATs in hemp.

Gene name	Gene ID	Chr	Chromosome location	Gene length	ORF length	Deduced protein				Subcellular location
				(bp)	(bp)	Size(aa)	MW(Da)	pI	GRAVY	
CsHATs-1	XP_030488713.1	1	5680290–5685571	5281	1320	442	51481.95	6.97	− 0.645	Cytoplasmic
CsHATs-2	XP_030494462.1	3	8294377–8298241	3864	1686	564	63325.71	8.72	− 0.355	Cytoplasmic
CsHATs-3	XP_030497419.1	4	68894034–68902579	8545	5153	1722	194421.46	8.29	− 0.716	Periplasmic
CsHATs-4	XP_030497420.1	4	68894034–68902579	8545	5117	1710	193196.17	8.29	− 0.718	Periplasmic
CsHATs-5	XP_030497421.1	4	68894034–68902519	8485	5093	1702	192317.07	8.29	− 0.721	Periplasmic
CsHATs-6	XP_030497422.1	4	68894034–68902579	8545	5006	1673	189408	8.33	− 0.731	Periplasmic
CsHATs-7	XP_030498501.1	4	91808612–91812568	3956	1613	541	60525.64	6.16	− 0.67	Cytoplasmic
CsHATs-8	XP_030498502.1	4	91809049–91812568	3519	1491	500	55662.22	6.18	− 0.635	Cytoplasmic
CsHATs-9	XP_030498504.1	4	91809111–91812568	3457	1324	444	49115.2	6.28	− 0.544	Cytoplasmic
CsHATs-10	XP_030510731.1	6	70330038–70345368	15330	5676	1898	214202.7	5.83	− 0.814	Cytoplasmic
CsHATs-11	XP_030484364.1	8	52452222–52454885	2663	1394	467	52065.25	5.13	− 0.204	OuterMembrane

Six CsHATs comprised 440–750 amino acids, and five CsHATs comprised 1700–1900 amino acids. The molecular weights (MWs) of CsHATs varied from 49115.2 to 214202.7 Da. *CsHATs-1* encoded the shortest protein, whereas *CsHATs-10* encoded the longest protein with the highest molecular weight of 214202.7 Da. The isoelectric points (PIs) varied from 5.11 to 8.72. Six CsHATs were detected in the cytoplasm.

### Phylogenetic analysis of CsHATs

**Fig. 2:**
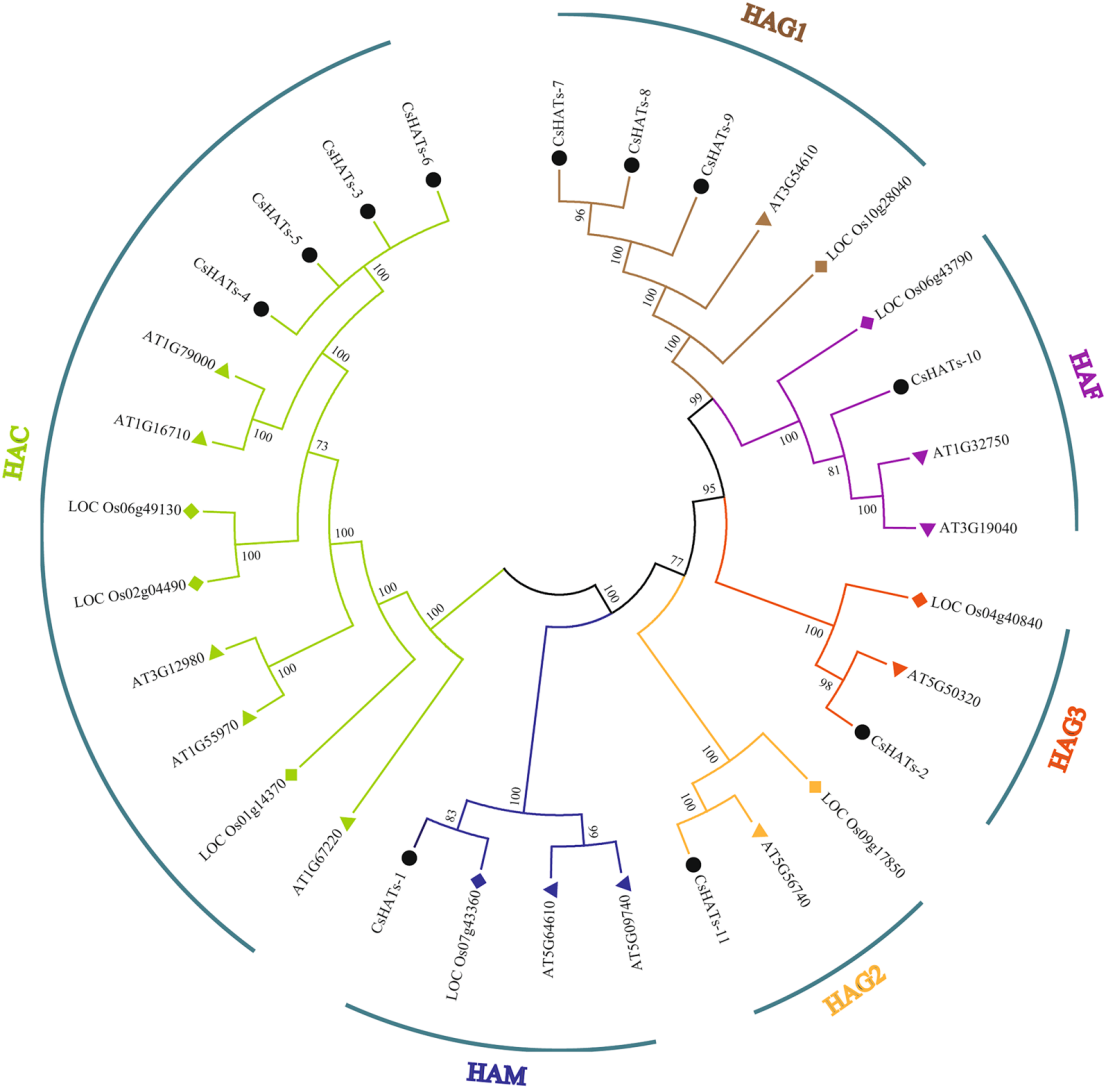
Phylogenetic tree of CsHATs from *Arabidopsis*, *Oryza sativa,* and hemp. A phylogenetic tree was constructed using the NJ (neighbour-joining) method. Each group is shown in different colours. Triangles represent *A. thaliana*, quadrangles represent *O. sativa*, and circles represent hemp

To understand the phylogenetic relationships among HATs from different species, we constructed a neighbor-joining (NJ) tree with 12 AtHATs, 8 OsHATs, and 11 CsHATs. As shown in Fig. 2, the HAT proteins of these species were divided into six categories, which shared a high homology. The HAC group was the largest, comprising 12 HAT proteins, including four CsHATs (CsHATs-3, CsHATs-4, CsHATs-5, and CsHATs-6), five AtHATs (AtHAC1, AtHAC2, AtHAC4, AtHAC5, and AtHAC12), and three OsHATs (Os06g49130, Os02g04490, and Os01g14370). The HAG1 group contained five HAT proteins, including three CsHATs (CsHATs-7, CsHATs-8, and CsHATs-9), one AtHAT (AtHAG1), and one OsHAT (Os10g28040). The HAG2 and HAG3 groups contained three HAT proteins each. The HAG2 group contained CsHATs-11, AtHAG2, and Os09g17850, whereas the HAG3 group contained CsHATs-2, AtHAG3, and Os04g40840. The HAF and HAM groups contained four HAT proteins. The HAF group contained CsHATs-10, AtHAF1, AtHAF2, and Os06g43790, whereas the HAM group contained CsHATs-1, AtHAM1, AtHAM2, and Os07g43360.

### Gene structures and conserved motifs of CsHATs

**Fig. 3:**
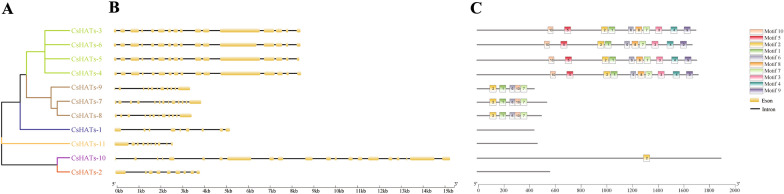
Phylogenetic relationships, gene structure, and conserved domains of histone acetyltransferase gene (*HATs*) in hemp. **A** Phylogenetic tree was constructed based on the sequences of CsHAT proteins using MEGA 7 software. The details of the clusters are shown in different colours. **B** Exon–intron structure of *CsHATs.* Yellow boxes indicate exons and black lines indicate introns. **C** Conserved domains of the HAT proteins in *Cannabis sativa*. Motifs numbered 1–10 are displayed in different coloured boxes

The comparison of gene structures provides insight into the evolution of the gene family. Thus, we analyzed the structures of the *CsHATs*. The positions and numbers of exons were significantly different for *CsHATs* across different phylogenetic groups and were relatively conserved among those within a group. The introns also varied significantly among the different phylogenetic groups (Fig. 3A, B). All genes contained introns and exons, and the number of introns ranged from 8 to 20, whereas those of exons ranged from 9 to 21 (Fig. 3B). Genes clustered in the same group shared similar gene structures.

The predicted amino acid sequences of the 11 CsHAT proteins were queried on MEME to characterize the putative motifs in the hemp HAT family. Ten motifs were predicted for these proteins, namely, motifs 1–10 (Fig. 3C). Members of the same group contained similar motifs, suggesting similar functions. Three CsHAT proteins did not contain any of the ten motifs, and eight CsHAT proteins contained varying numbers of motifs. All 10 motifs were found in CsHATs-3, CsHATs-4, CsHATs-5, and CsHATs-6. Five motifs (motif1, motif2, motif6, motif7, and motif10) were found in CsHATs-7, CsHATs-8, and CsHATs-9. Motif2 was found in CsHATs-10. These results indicated conserved motif compositions and similar gene structures among HAT members in the same group. Together with the phylogenetic analysis results, the reliability of the group classifications was validated. The motif structure is shown in Additional file 3: Figure S1.

### Analysis of Cis-acting elements in promoters of CsHATs

**Fig. 4:**
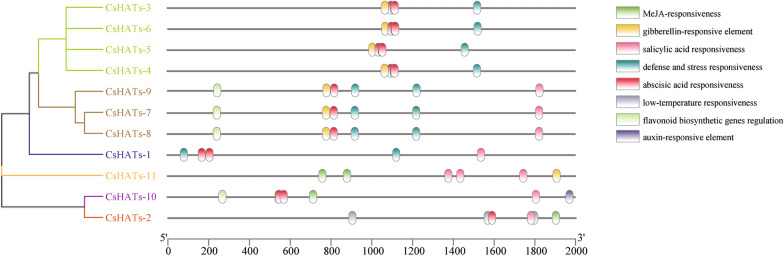
Prediction of cis-acting elements in the *CsHAT* promoters. The phylogenetic tree was constructed based on the sequences of CsHAT proteins using MEGA 7 software. The details of the clusters are shown in different colours. The type, quantity, and position of the elements in the *CsHAT* promoters are shown

To further study the potential regulatory mechanisms of CsHATs during stress responses, 2000 bp upstream sequences from the translation start sites of *CsHATs* were retrieved from TBtools and submitted to PlantCARE to detect the *cis*-elements. Eight stress response elements, namely, salicylic acid, abscisic acid, jasmonic acid, auxin, gibberellin, flavonoid biosynthesis gene regulatory elements, defense and stress response elements, and low-temperature response elements were analyzed (Fig. 4). Some stress-response-related *cis*-elements were detected in the promoters of *CsHATs*. These hormone regulatory elements widely exist in *CsHAT* promoters, and the auxin-response element unique to *CsHAT-10*. Defense and stress response elements existed in the promoters of all *CsHATs,* except for *CsHATs-2*, *CsHATs-10*, and *CsHATs-11*. The flavonoid biosynthesis gene regulation elements were present in four *CsHATs* (*CsHATs-7*, *CsHATs-8*, *CsHATs-9*, and *CsHATs-10*); low-temperature response elements were only found in *CsHATs-2*. The *cis*-element analysis illustrated that *CsHATs* could respond to various stresses.

### Collinearity analysis for CsHATs

**Fig. 5:**
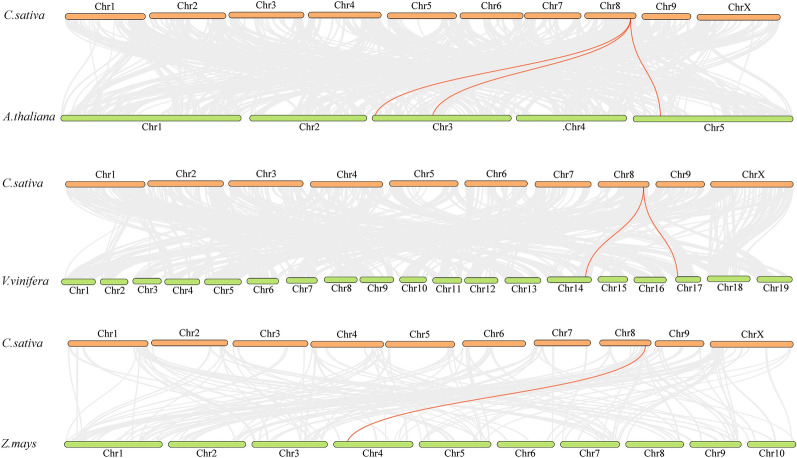
Synteny analysis of *HATs* between hemp and three representative plant species (*Arabidopsis*, grape, and maize). Grey lines in the background indicate the collinear blocks within hemp and other plant genomes, whereas the red lines highlight the syntenic *HAT* pairs

We constructed three comparative syntenic maps of hemp associated with three representative species, two dicots (*Arabidopsis* and grape), and one monocot (maize) to further infer the phylogenetic mechanisms of *CsHATs* (Fig. 5). Three genes in *Arabidopsis* were collinear with *CsHATs*; likewise, two in grapes and one in maize were observed. The number of orthologous pairs between hemp and the three species (*Arabidopsis*, grape, and maize) was 3, 2, and 1. This finding indicated that these orthologous pairs may have already existed prior to the divergence of dicotyledonous and monocotyledonous plants.

### Expression pattern analysis for CsHATs

**Fig. 6:**
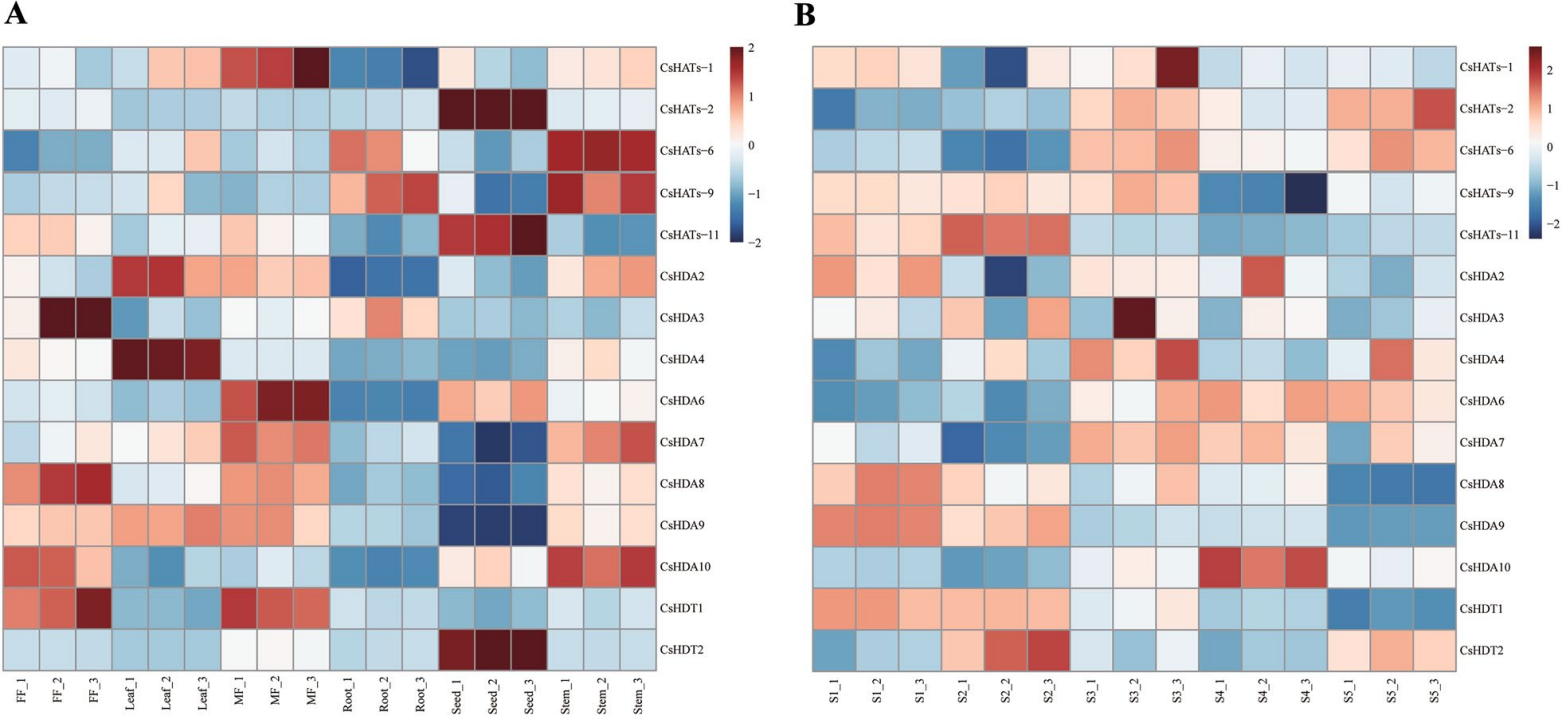
Expression patterns of *HATs* and histone deacetylase genes (*HDACs*) in hemp. **A** Expression of *HATs* and *HDACs* across different organs. **B** Expression of *HATs* and *HDACs* at different inflorescence developmental stages. Heat maps reflect the fragments per kilobase of transcript per million mapped fragments (FPKM) values of *HATs* and *HDACs*. Colours from red to blue indicate high to low expression

Previously, we obtained RNA-seq data containing different organs and inflorescence developmental stages of hemp (S1: apical meristem, female flowers absent; S2: the female flowers appear and stigma is white; S3: the stigma is orange when pollination is complete; S4: when the seeds are green and not yet ripe, and S5: when the seeds are mature and brown) (unpublished). Based on the RNA-seq data, we examined the expression patterns of *CsHATs*, some of which showed similar expression patterns across organs (Fig. 6A). *CsHATs-6* and *CsHATs-9* were highly expressed in roots and stems but were rarely detected in seeds, female flowers, and male flowers. *CsHATs-2* was highly expressed only in the seeds. The expression of some genes showed significant trends at different developmental stages (Fig. 6B). For example, the levels of *CsHATs-2* and *CsHATs-6* expression gradually increased with inflorescence development, whereas those of *CsHATs-1*, *CsHATs-9,* and *CsHATs-11* declined towards the subsequent developmental stages of inflorescence, involving seed development and maturation.

The levels of deacetylation gene expression were analyzed in hemp. According to the members of the *CsHDAC* gene family screened in a previous study [37], 10 *CsHDACs* were identified based on our transcriptomic data. These *CsHDACs* were highly expressed in different hemp organs, especially in almost all female flowers, male flowers, and stems (Fig. 6A). The expression of *CsHDA4*, *CsHDA6*, *CsHDA7, CsHDA10*, and *CsHDT2* gradually increased from S3–S5 (Fig. 6B). These results indicated that *CsHATs and CsHDACs* were widely and differently expressed in different organs and developmental stages of hemp, and they may synergistically affect protein acetylation in these organs and across developmental stages.

### Expression patterns of cannabinoid biosynthesis genes and PTM of histones in hemp

**Fig. 7:**
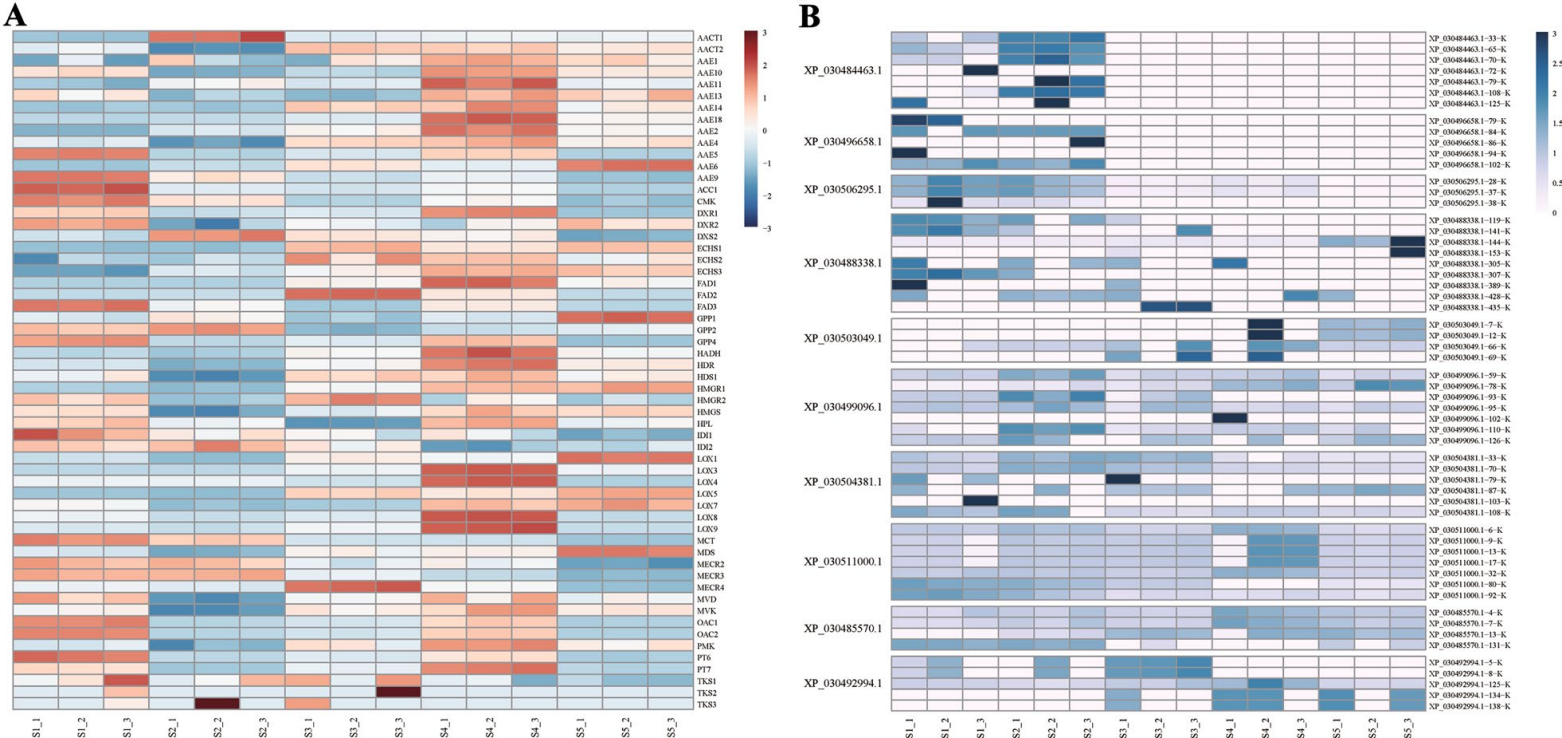
The expression patterns of the cannabinoid synthesis pathway genes and acetylation patterns of different histone lysines in hemp. **A** Expression of cannabinoid synthesis pathway genes at different inflorescence developmental stages. Heat maps reflect the fragments per kilobase of transcript per million mapped fragments (FPKM) values of cannabinoid synthesis pathway genes. Colours from red to blue indicate high to low expression. **B** Acetylation pattern of different histone lysines across inflorescence developmental stages of hemp

Cannabinoid content shows significant changes during inflorescence development [38]. We detected the expression of cannabinoid biosynthesis pathway genes based on RNA-seq data. As shown in the heatmap (Fig. 7A), these genes were differentially expressed across different inflorescence developmental stages. For example, *LOX4*, *LOX8*, and *LOX9* were expressed only at S4. Some genes were expressed at most stages, such as *AACT2*, *AAE4*, *HDS1* and *MVK* in S1, S3, S4, and S5. Some genes, such as *PT6*, *GPP4*, *AAE5*, *OAC1*, and *OAC2*, were expressed only in S1 and S4.

HATs and HDACs can catalyze histone acetylation to regulate gene expression [39]. Thus, we examined the acetylation patterns of histones during the five inflorescence developmental stages (Fig. 7B) (laboratory self-test, unpublished). Ten histones were acetylated during the inflorescence development. As shown in the heat map, XP_030484463.1, XP_030496658.1, XP_030506295.1, and XP_030488338.1 were highly acetylated in S1 and S2, whereas XP_030503049.1 was highly acetylated in S4 and S5. XP_030499096.1, XP_030504381.1, XP_030485570.1, and XP_030492994.1 were highly acetylated throughout all stages. These results indicated that histone acetylation occurs widely during inflorescence development and may affect the expression of cannabinoid biosynthesis pathway genes. In addition, we found that the expression of *CsHATs* and *CsHDACs* genes were significantly associated with cannabinoid synthesis genes. As shown in Additional file 4: Figure S2, *CsHATs* (*CsHATs-1*, *CsHATs-2*, *CsHATs-6*, *CsHATs-9*, and *CsHATs-11*) and *CsHDACs* (*CsHDA2*, *CsHDA3*, *CsHDA4*, *CsHDA6*, *CsHDA7*, *CsHDA8*, *CsHDA9*, *CsHDA10*, *CsHDT1*, and *CsHDT2*) were positively correlated with some cannabinoid synthesis genes. It indicated that *CsHATs and CsHDACs* may regulate the expression of cannabinoid synthesis genes.

### Effect of histone acetylation inhibitor treatment on cannabinoid biosynthesis gene expression and contents of CBD and CBG

**Fig. 8:**
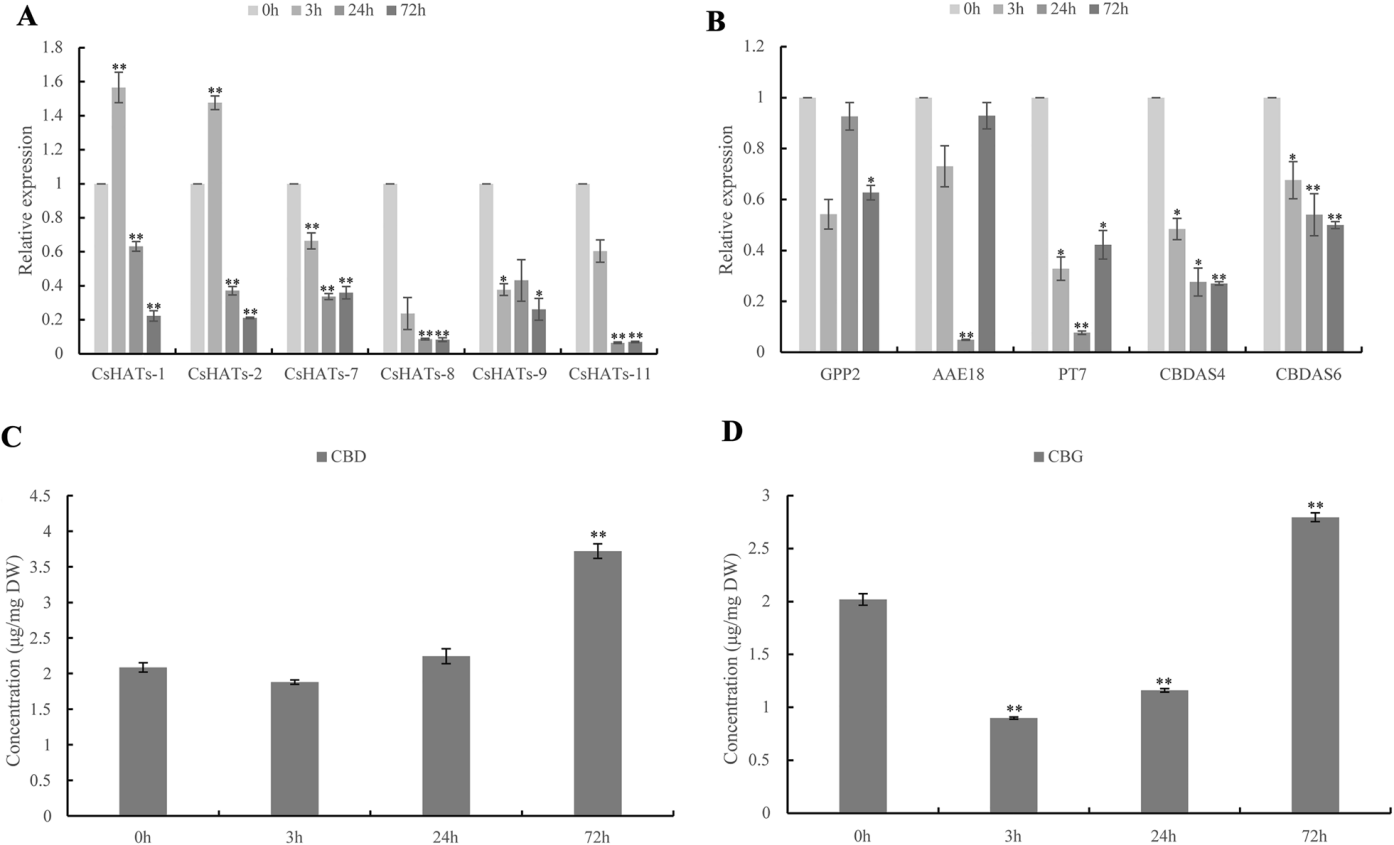
Relative expression of genes and the contents of CBD and CBG after PU139 treatment. **A** Levels of *CsHAT* expression after inhibitor treatment (measured by qRT-PCR). **B** Gene expression associated with cannabinoid synthesis after inhibitor treatment (measured by qRT-PCR). Data were normalised with the levels of *EF1α*, and vertical bars indicate the standard deviation. **C**–**D** Contents of CBD and CBG after PU139 treatment. Vertical bars indicate the standard deviation. (**P < 0.01; *P < 0.05)

We treated hemp inflorescences with a histone acetylation inhibitor (PU139) to further investigate whether histone acetylation could affect cannabinoid biosynthesis. PU139 effectively inhibits the expression of the *CsHATs*. The levels of six *CsHATs* (*CsHATs-1*, *-2*, *-7*, *-8*, *-9*, and *-11*) were downregulated at different time points after inhibitor treatment (Fig. 8A). The expression of cannabinoid biosynthesis pathway genes, *AAE18*, *GPP2*, *PT7*, *CBDAS4*, and *CBDAS6* was generally downregulated upon exposure to PU139 for different times (Fig. 8B). We also determined the CBD and CBG contents before and after treatment. The CBD content declined only 3 h after inhibitor administration (Fig. 8C). The CBG content decreased at 3 h and 24 h after inhibitor treatment and increased at 72 h (Fig. 8D). Therefore, inhibition of histone acetylation resulted in the inhibition of cannabinoid biosynthesis pathway gene expression and further decreased CBD and CBG contents in hemp inflorescence.

## Discussion

In this work, we identified 11 HATs, of which 64% was found in the cytoplasm and 36% in the periplasm. The HAT gene family has been identified and characterized in several plants, involving 12 genes in *A. thaliana*, [40] 8 genes in *O. sativa*, [41] 31 genes in *T. aestivum*, [33] 7 genes in *Vitis vinifera*, [42] and 32 genes in tomato [43]. These results indicated that the numbers of HATs were diverse. According to previous research on HATs, we found that the majority of *CsHATs* were B-type HATs, and acetylate histones were in the cytoplasm [6, 44]. The HAT genes could be divided into six families: HAC, HAF, HAM, HAG1, HAG2, and HAG3 [33]. Gene family refers to a collection of genes with similar function or structure, and these genes usually have similar sequence characteristics. Genes in a gene family may or may not have the same motifs. Motif is a short sequence in a gene or protein sequence that has a specific function. And it is not directly related to the gene family. 11 CsHATs belong to histone acetyltransferases but it was be divided into six subgroups. The same groups were usually found to share a similar motif composition. The similar motif arrangements among proteins within subgroups indicated that the protein architecture is conserved within a specific subgroup. For example, pineapple WRKY proteins and potato Hsp20 proteins, subgroups have similar motifs, but not all family proteins have the same motif [52, 53]. Motif analysis found that *CsHATs-1*, *CsHATs-2*, and *CsHATs-11* did not contain any motifs. However, HAC and HAG1 subgroups contained similar motif. This observation revealed that different groups of the phylogenetic tree presented different motifs. Previous study had found that there were gene deletion events in gene families. For example, Hsp20 in chili lacks the CIV, CV, and CVIII subfamilies [54]. Gene structure analysis and motif analysis also indicated that *CsHATs-1*, *CsHATs-2* and *CsHATs-11* may also be lost gradually during evolution. Subsequently, within 2000 bp-upstream sequences from the translation start sites of the *CsHATs*, stress-related cis-acting elements and hormone-related elements were identified. This finding suggested that *CsHATs* played a crucial role in resistance to adversity [6].

The length of *CsHATs-11* is 2663 bp. It is located on chr8, and only this gene has a collinearity relationship with *Arabidopsis*, grape and maize. And there are 3 pairs, 2 pairs and 1 pair of collinear pairs between hemp and these three species (*Arabidopsis*, grape and maize), respectively. This finding indicated that these collinear pairs may have already existed prior to the divergence of dicotyledonous and monocotyledonous plants. For example, some collinear gene pairs identified between pineapple and rice, maize, bananas *Arabidopsis* and grape were found, which may indicate that these orthologous pairs may already exist before the ancestral divergence [52].

Although *HATs* play an important role in growth, development processes, and stress responses, the specific functions of *HATs* in hemp remain unknown. Reports showed that *AtHAC1* regulates factors upstream of FLC at flowering time through epigenetic modification and interacts with the tomato heat stress transcription factor HsfB1 [45]. Therefore, the *CsHAT-3/4/5/6* homologs with *AtHAC1* may influence flowering in hemp. We hypothesize that the closest *CsHATs-1* orthologs may perform the same function given that *AtHAM1* and *AtHAM2* are involved in the formation of male and female gametophytes [46]. *AtHAG1* plays a crucial role in cell differentiation and leaf and flower organ formation [47]. *CsHATs-7/8/9* may share similar function with *AtHAG1* according to phylogenetic analysis. These results suggest that *CsHATs-7/8/9* may have a role in plant development. Histone H4K12 is acetylated by *AtHAG2*. [48] Accordingly, the *AtHAG2* homolog *CsHATs-11* may also perform the same function. *AtHAG3* can regulate plant response to ABA [49]. *CsHATs-2* may also have similar functions because it clusters together with *AtHAG3* in the phylogenetic tree. These analyses strongly suggest that *CsHATs* play important roles in the growth and development of hemp. More detailed characterization of their functions can provide guidance for the cultivation of superior hemp varieties.

The expression patterns of *HATs* in different organs have been described in many species, including *Triticum aestivum*, cotton, and foxtail millet [33, 50, 51]. For example, *TaHAT* genes were highly expressed in wheat leaves [33]. *GhHATs* were widely expressed in the vegetative (root, stem, and leaf) and reproductive (torus, petal, stamen, pistil and calyx) parts [50]. *SiHAT2* was highly expressed in leaves and stems, but the transcript levels of *SiHAT4*, *SiHAT15*, and *SiHAT18* were very low [51]. In addition, Yang et al. found that *CsHDACs* were expressed in roots, stems, leaves, and flowers to different degrees [37]. *CsHATs* exhibited diverse expression patterns in the investigated organs during inflorescence development in our study. Meanwhile, *CsHATs* transcript levels were abundant in male flowers, roots, stems, leaves, and seeds. The transcription levels of *CsHDACs* in different organs were also different. Therefore, the expression of *HATs* and *HDACs* genes were spatiotemporal specificity. Interestingly, the transcript levels of cannabinoid synthesis genes were expressed differently, as well as the acetylation levels of different histones during the different stages of hemp. We predict that the acetylation of *CsHATs* may be related to the synthesis of cannabinoid.

The relative expression levels of *HATs* change significantly under various abiotic stresses [6]. Recent studies have established the link of histone acetylation and abiotic stresses [33, 50]. For example, in *Triticum aestivum*, cold exposure represses the expression of *TaHATs* [33]. The expression of *GhHAC1502* strongly increased in response to NaCl, whereas the expression of *GhHAC1503* was strongly induced by Zn [50]. Our expression data suggest the functional diversity and specificity among different subgroups of *CsHATs* in response to inhibitors (PU139). These observations suggest that *CsHATs* likely play an important role in hemp plant adaptation to various abiotic stresses. Furthermore, the genes involved in cannabinoid synthesis were changed under inhibitor treatment. This finding suggests that different *CsHATs* and cannabinoid synthesis genes may work together in response to specific stimuli. The content of CBD and CBG were low and undetected before the female flowers appearing in our study, and the relationship of the expression of *CsHATs* and *CsHDACs* genes and the production of cannabinoids were not analyzed.

In this study, 11 *HATs* in hemp were identified based on their genomic information. These 11 *HATs* were divided into six main groups, and genes in the same group showed highly similar exon–intron structures and motif composition. The spatiotemporally specific expression of *CsHATs* was shown in different organs during inflorescence developmental stages of hemp. In addition, the expression of *CsHATs* and the contents of CBD and CBG declined in the samples treated by inhibitor. These results indicated that *CsHATs* regulated cannabinoid biosynthesis. Our study systematically analyzed the characteristics and expression patterns of *HATs* and provided genetic information to enhance understanding of the biosynthesis of cannabinoids, as well as valuable resources for breeding high-quality hemp varieties, such as those with high CBD, low HTC, and resistance to diverse stresses. At present, the role of *CsHATs* in the process of cannabinoid biosynthesis has been preliminarily explored in our paper. Next, we will analyze the expression profile of CBD/CBG biosynthetic genes between samples treated and untreated with PU139, and further verify the function of *CsHATs*. These will provide more direct evidence for the role of *CsHATs* in cannabinoid biosynthesis of hemp.

## Supplementary Information

**Additional file 1: Table S1.** Genes and their primer sequesnces for qRT-PCR.

**Additional file 2: Table S2.** The acquisition parameters of each compound.

**Additional file 3: Figure S1.** The structures of ten motifs in hemp.

**Additional file 4: Figure S2.** Correlation analysis of CsHATs and CsHDACs with cannabinoid synthesis genes.

## Data Availability

The data analyzed during this study can be obtained from the corresponding author on reasonable request.
