# Supplementary figures and images for: Identification of histone acetyltransferase genes responsible for cannabinoid synthesis in hemp
Source: Chin Med. 2023 Feb 13;18:16. doi: 10.1186/s13020-023-00720-0 (PMC9926835; doi:10.1186/s13020-023-00720-0)

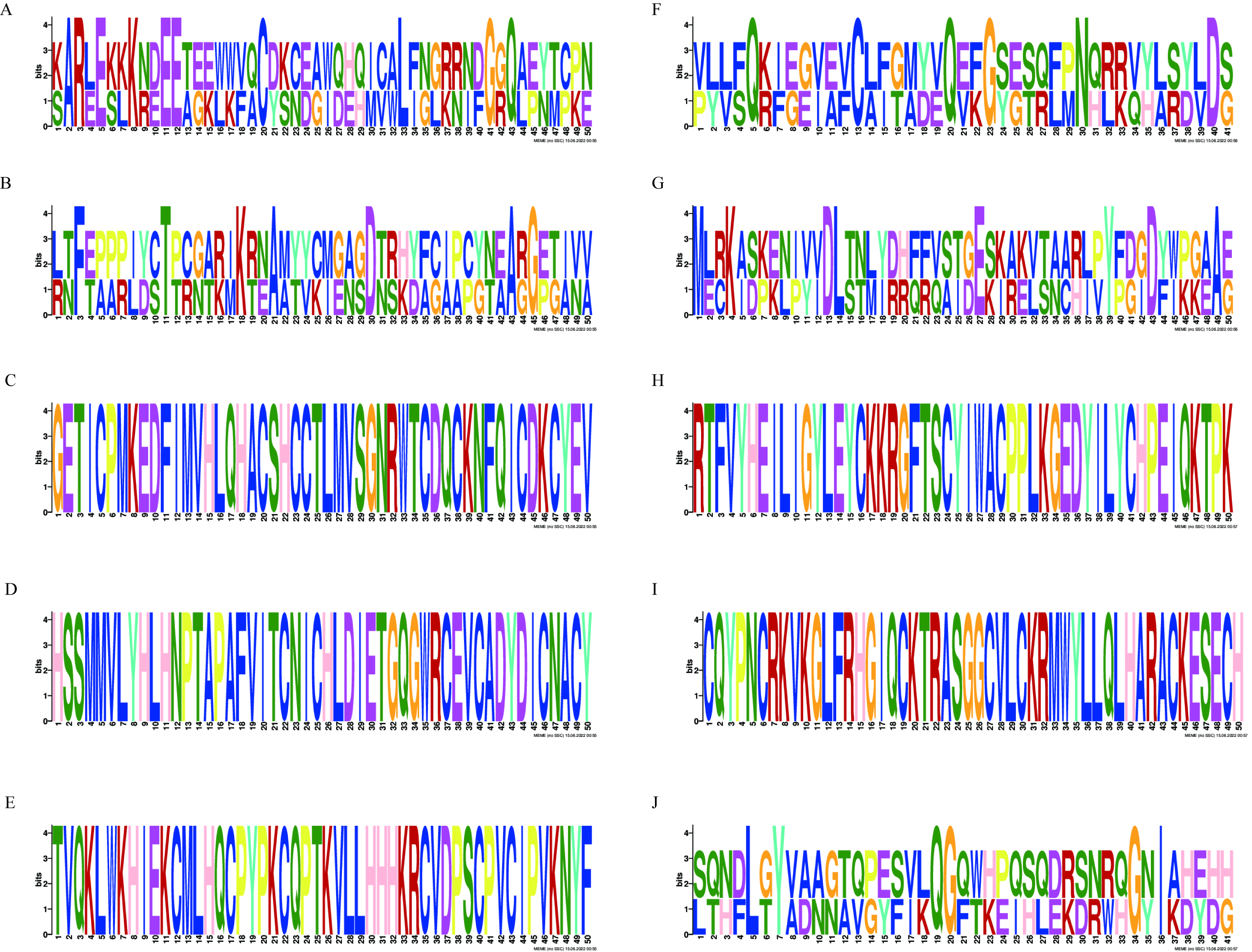

Supplement: Supplementary file 3 — Additional file 3: Figure S1. The structures of ten motifs in hemp. [file 13020_2023_720_MOESM3_ESM.tif]

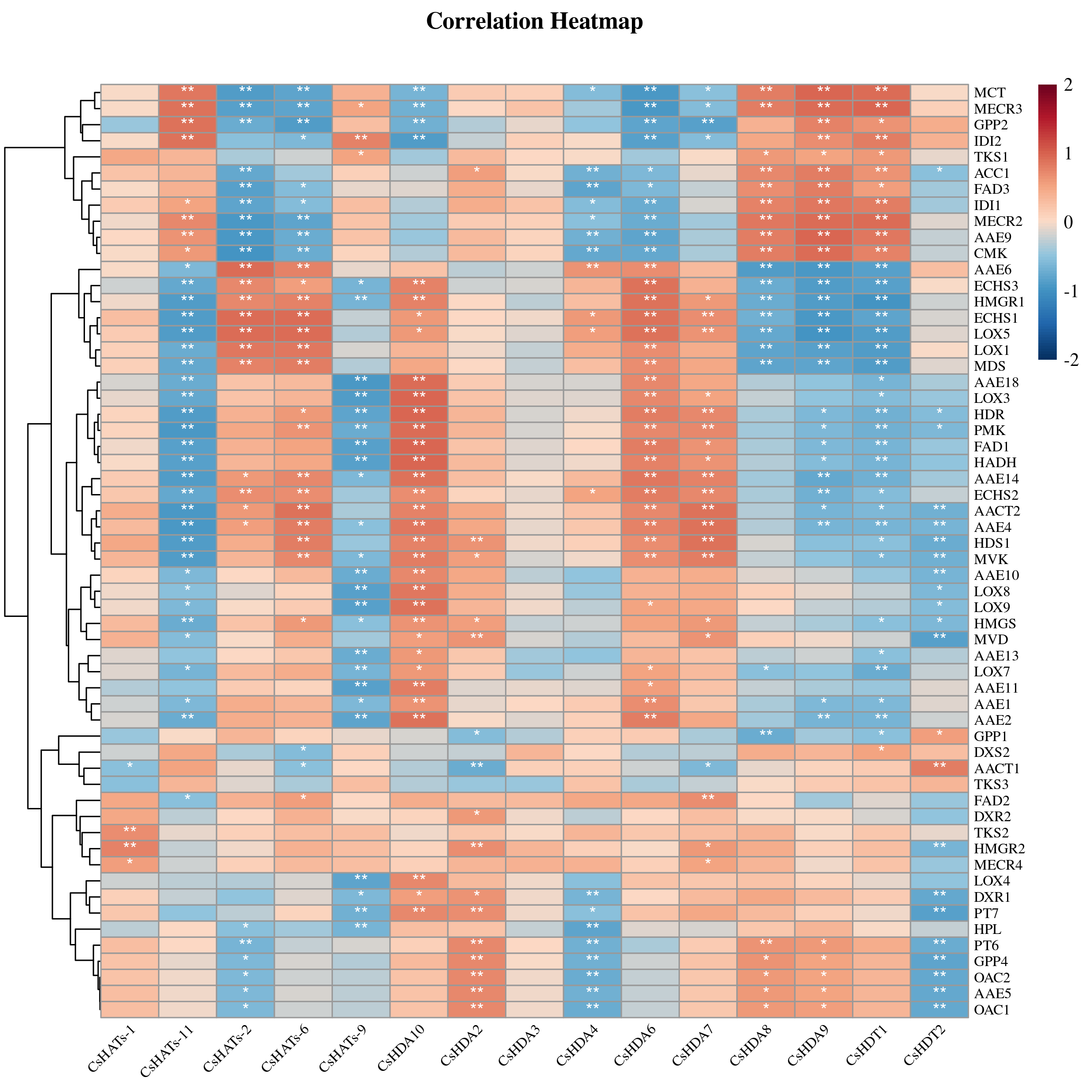

Supplement: Supplementary file 4 — Additional file 4: Figure S2. Correlation analysis of CsHATs and CsHDACs with cannabinoid synthesis genes. [file 13020_2023_720_MOESM4_ESM.tif]
